# Modulation of Gut Microbiota, Intestinal Physiology, and Digestive Enzyme Levels by Duo-Strain Probiotics in African Catfish (*Clarias gariepinus*) Challenged With *Aeromonas hydrophila*

**DOI:** 10.1155/anu/6624613

**Published:** 2025-11-21

**Authors:** Nurul Aini, Sri Puji Astuti Wahyuningsih, Divany Hunaimatul Achhlam, Fatimah, Muhammad Hilman Fu'adil Amin, Hoang Dang Khoa Do

**Affiliations:** ^1^Doctoral Mathematics and Natural Sciences Study Program, Faculty of Science and Technology, Universitas Airlangga, Surabaya, Indonesia; ^2^Department of Agricultural Technology, Universitas KH. A. Wahab Hasbullah, Jombang, Indonesia; ^3^Department of Biology, Faculty of Science and Technology, Universitas Airlangga, Surabaya 60115, Indonesia; ^4^RG Developmental Biology and Biomedical Science, Universitas Airlangga, Surabaya, Indonesia; ^5^University CoE Research Center for Bio-Molecule Engineering, Universitas Airlangga, Surabaya, Indonesia; ^6^NTT Hi-Tech Institute, Nguyen Tat Thanh University, Ho Chi Minh City, Vietnam

**Keywords:** *Aeromonas hydrophyla*, catfish, digestive enzyme, gut heath, metagenomic, probiotic supplementation

## Abstract

This study aimed to determine the effect of dual-strains probiotic (DSP) consisting of *Lactobacillus casei* and *Bacillus subtilis* on bacterial metagenomic profile, gut physiology, and digestive enzyme levels of African catfish (*Clarias gariepinus*) infected by *Aeromonas hydrophila*. The ratio between *L. casei* and *B. subtilis* was 1:1 each with a density of 10^8^ CFU/mL. Catfish (*n* = 8 fish per tank, three replicates per treatment) were fed diets supplemented with 0%, 5%, 10%, or 15% DSP for 42 days. On the 35th day, selected groups were intraperitoneally challenged with *A. hydrophila* at a dose of 0.1 mL × 10^8^ CFU/mL. The observed parameters included bacterial counts and microbial profile in the gastrointestinal tract (analyzed using next-generation sequencing [NGS]), gut physiology, and digestive enzyme levels (amylase, lipase, and protease). The results showed that DSP supplementation increased both the abundance and diversity of gastrointestinal microbes, elevated digestive enzyme levels, and enhanced the number of goblet cells in the intestinal lining. The dominant microbial phyla observed in the control group were Fusobacteria and Pseudomonadota.

## 1. Introduction

Aquaculture is the largest and fastest-growing food production sector globally, representing a major potential to meet the increasing demand for aquatic food [1]. Among its outputs, fisheries have become an essential source of nutrition, as well as a vital source of livelihood and economic benefit for individuals involved in fish farming, harvesting, processing, and marketing activities [2]. One of the primary commodities in the fisheries sector is catfish (*Clarias* sp.), which is widely cultivated due to its simple rearing process, high economic value, and adaptability to various types of feed and limited farming space [3–5].

To maintain stable fish stocks and meet the basic needs of the fisheries sector, effective fisheries management is essential to address various challenges such as poor water quality, high feed costs, and pathogenic infections [6–8]. One of the most common pathogenic bacteria affecting catfish is *Aeromonas hydrophila* [9]. *A. hydrophila* causes Motile Aeromonas Septicemia (MAS), a disease that initially infects the external parts of the fish and subsequently spreads systemically through the circulatory system [10]. It can lead to conditions such as dermatitis, orbital cellulitis, and ocular rupture.

Once inside the fish, the pathogen invades blood vessels and internal organs, leading to tissue and organ damage. Infected catfish often experience high mortality rates, with death occurring in 80%–100% of cases within approximately 1 week of infection [11]. In addition to systemic infection, *A. hydrophila* can cause intestinal damage, including necrosis of the epithelial layer in the intestines of *Clarias gariepinus*. This damage also results in a reduction of goblet cells, which serve as important components of the intestinal barrier defense [12]. Furthermore, the pathogen can induce histopathological damage to the pancreas, thereby impairing its function and reducing the secretion of digestive enzymes [13].

The prevalence of infectious diseases in catfish farming has traditionally been addressed through the use of antibiotics [14, 15]. However, the antibiotics commonly used by catfish farmers are synthetic and may contaminate the aquatic environment [16]. Moreover, the continuous use of antibiotics and chemical agents can negatively impact human health, fish growth, and surrounding ecosystems [17]. Therefore, alternatives to antibiotics, such as probiotics, are increasingly being explored [18].

A probiotic is a single- or multispecies microorganism that provides beneficial effects to its host when administered in sufficient quantities, such as by enhancing fish growth and improving health status during aquaculture operations [19, 20]. Consequently, probiotics have long been marketed as aquatic or feed additives for a variety of aquaculture species [21, 22]. The most commonly used probiotic bacteria in aquaculture—many of which are indigenous—belong to the genera *Bifidobacterium*, *Enterococcus*, *Bacillus*, *Lactobacillus*, *Lactococcus*, *Leuconostoc*, *Pediococcus*, and *Weissella*, all of which have been isolated from the intestinal tracts of various fish species [23–25].

*Lactobacillus casei* and *Bacillus subtilis* are probiotic bacteria that offer greater advantages compared to other bacterial strains and play an important role in aquaculture [26]. Previous studies have demonstrated that the combination of these two strains can improve growth performance and enhance the immune response in catfish challenged with *A. hydrophila* [27]. Specifically, *L. casei* is resistant to bile salts and low pH, allowing it to survive in both the intestinal tract and stomach. As a result, it positively influences the growth and microbial biodiversity of the host's gastrointestinal system [28, 29]. The *Bacillus* genus has been widely applied in aquaculture and is known to benefit fish health [30], as these bacteria can produce extracellular enzymes that support digestion and can withstand environmental stress conditions such as limited water availability and extreme temperatures [31].

The fish digestive tract is a highly complex ecosystem that harbors a diverse array of microflora, which play a vital role in nutrient provision for the host [32]. In addition, the microflora can enhance the immune system by inhibiting the growth and proliferation of pathogenic microbes, regulating the development of intestinal organs, and producing essential vitamins beneficial to the host [33]. Studies on gut microflora have provided insights into the structure of bacterial populations, allowing for a deeper understanding of how pathogenic microorganisms develop in the gut environment [34].

Various types of beneficial bacteria are intentionally incorporated into fish feed to enhance growth performance and improve feed efficiency [35]. Beyond assisting in food digestion, gut microflora also contributes to the fermentation of substrates, including metabolic waste products.

Similar effects of dual-strain probiotics (DSP) have been reported in other freshwater fish species, such as grass carp (*Ctenopharyngodon idella*). A study by Luo et al. [34] demonstrated that the use of DSP—a combination of *L. plantarum* and *B. subtilis*—during feed supplementation significantly increased growth, digestive enzyme production (including total protease, α-amylase, lipase, and trypsin), and antioxidant capacity (such as superoxide dismutase, malondialdehyde, and catalase). Furthermore, the diversity of gut microbiota in *Ctenopharyngodon idella* increased after 30 days of rearing, with dominant bacterial groups, including Bacteroidetes, Firmicutes, Fusobacteria, and Proteobacteria. In another study, Wang et al. [28] reported that the administration of *L. casei* K17 enhanced growth rate, nutrient absorption and digestion, nonspecific immune response, intestinal microbiota composition, and overall metabolism in *Micropterus salmoides*.

Hamka et al. [36] demonstrated that the combination of probiotic bacteria *Bacillus megaterium* PTB 1.4 and *Pediococcus pentosaceus* E2211 enhanced protease and amylase activity in the gastrointestinal tract of catfish (*Clarias* sp.). In another study, the addition of a multispecies probiotic formulation consisting of *B. subtilis*, *L. plantarum*, *Enterococcus faecium*, and *Saccharomyces cerevisiae* had a positive effect on *Pangasianodon hypophthalmus*. The results showed that probiotic supplementation increased the activity of digestive enzymes (lipase, amylase, and protease) as well as antioxidant enzyme activity [37]. *L.casei* has also been shown to significantly improve the composition of intestinal microbiota, thereby inhibiting pathogenic bacterial infections and enhancing intestinal barrier defenses, including increased mucin secretion by goblet cells [38].

The addition of two or more probiotic strains to fish feed is considered more beneficial than using a single strain, as they may work synergistically to provide enhanced effects [39]. Therefore, in this study, two probiotic strains *L. casei* and *B. subtilis* were incorporated into fish feed to evaluate their effects on intestinal microbial diversity, gut physiology (as indicated by the number of goblet cells), and the secretion of gastrointestinal enzymes.

## 2. Materials and Methods

### 2.1. Ethics Statement

The study was conducted in accordance with the ethical guidelines approved by the Animal Care and Use Committee (ACUC), Faculty of Veterinary Medicine, Universitas Airlangga (Ethical Approval Number: 2.KEH.073.05.2023).

### 2.2. Preparation of Experimental Fishes and Probiotic Strains

Catfish (*C. gariepinus*) with lengths ranging from 18 to 21 cm and weights between 75 and 90 g were immersed in a methylene blue solution (one drop per 25 L of water) for 7 days to acclimate to the new environment before the start of the experiment. During the acclimation period, the fish were fed commercial feed containing 33% protein, produced by PT Matahari Sakti Prima Feed LP-1 SP (Indonesia). Subsequently, the fish were randomly distributed into aerated aquaria at a density of eight fish per 70 L aquarium. They were fed twice daily at a feeding rate of 5% of body weight and reared for 42 days, following a previous study [31].

Bacterial isolates of *L. casei* FNCC 0090 and *B. subtilis* 0059 were obtained from the Center for Food and Nutrition Studies, Universitas Gadjah Mada, Yogyakarta, Indonesia. The *L. casei* strain was cultured in De Man, Rogosa, and Sharpe Broth (MRSB; HiMedia, USA), while *B. subtilis* and *A. hydrophila* were cultured in Nutrient Broth (NB; HiMedia, USA). All bacteria were incubated for 48 h at 35°C, a temperature selected because it represents the optimal growth range for both *L. casei* and *B. subtilis* as reported in previous studies, while also approximating the gut environment of tropical freshwater fish (28–32°C). This condition ensured high bacterial viability before incorporation into feed, with cultures reaching a density of 10^8^ CFU/mL, determined using a spectrophotometer and the total plate count (TPC) method. The *L. casei* and *B. subtilis* cultures were combined in a 1:1 ratio to formulate the DSP. The probiotic mixture was sprayed onto commercial feed at concentrations of 0%, 5%, 10%, and 15% (based on feed weight). This corresponded to approximately 1 × 10^7^–10^8^ CFU/g of feed. Considering the feeding rate of 5% of body weight (≈3.5–4.5 g feed per fish/day), each fish received an estimated intake of 1 × 10^8^−10^9^ CFU/day. The feed was then homogenized and allowed to ferment in a closed container under aerobic conditions at 28 ± 2°C and ambient humidity (65%–70%) for 18–24 h [27]. To confirm viability after fermentation, the probiotic counts were verified using the TPC method, showing that the bacteria remained viable within the target range.

To induce infection, *A. hydrophila* (0.1 mL × 10^8^ CFU/mL) was administered intraperitoneally to selected catfish on the 35th day of the rearing period, following the method described in a previous protocol [27]. In this study, DSP-supplemented feed was administered to both infected and noninfected fish. The treatment groups were as follows: (A) 0% DSP, infected; (B) 5% DSP, infected; (C) 10% DSP, infected; (D) 15% DSP, infected; (E) 0% DSP, noninfected; (F) 5% DSP, noninfected; (G) 10% DSP, noninfected; (H) 15% DSP, noninfected. Each treatment group was conducted in triplicate. Descriptions of treatment groups and experimental replications are in Table 1.

### 2.3. Isolation and Enumeration of Culturable Intestinal Microbiota

Prior to microbial isolation, fish were fasted for 24 h. The intestines were then collected and stored at −80°C until further processing. For each treatment group, intestines from three individual fish were sampled. Approximately 10 g of intestinal tissue was homogenized with 90 mL of 0.85% physiological saline solution. The resulting mixture was plated in duplicate onto sterile Petri dishes containing Nutrient Agar (Himedia) and incubated at 37°C for approximately 48 h. Following incubation, bacterial colonies were observed, recorded, and quantified as colony-forming units per milliliter (CFU/mL).

### 2.4. Isolation of Gut Bacterial DNA for Metagenomic Analysis

Total genomic DNA from catfish intestinal samples was extracted using the Quick-DNA Magbead Plus Kit (Zymo Research, D4082). DNA quality and concentration were assessed using gel electrophoresis, a NanoDrop spectrophotometer, and a Qubit fluorometer. PCR amplification was performed using KOD-Multi & Epi-TM (Toyobo, KME-101), targeting the V1–V9 regions with an expected fragment length of 1500 bp. The primers used were universal primers 27F and 1492R. Subsequently, 2 µL of PCR products were analyzed via 1% TBE agarose gel electrophoresis. Library preparation was carried out using the Native Barcoding Kit 96 V14 (Oxford Nanopore Technologies MinION, SQK-NBD114−96). Nanopore sequencing was conducted using MinKNOW software version 23.04.5. Base calling was performed with Guppy version 6.5.7 using the high-accuracy model [40].

FASTQ file quality was visualized and filtered using Nano-Plot and Nano-Filt, respectively [41, 42]. Filtered reads were classified using the Centrifuge classifier [43]. Bacterial and archaeal indices were generated using the NCBI 16S RefSeq database (https://ftp.ncbi.nlm.nih.gov/refseq/TargetedLoci/). Downstream analysis and visualization were performed using Pavian, Krona Tools, and RStudio (R version 4.2.3; https://www.R-project.org/). Dissimilarity matrices for microbial communities in catfish intestines under various DSP treatments and *A. hydrophila* infection were calculated using relative read abundance (RRA) with the Bray–Curtis distance method.

### 2.5. Observation of Intestinal Physiology

The intestinal organs were rinsed with normal saline solution, then cut into two sections and placed in 10% neutral buffered formalin (NBF) solution. Histological preparation of the intestinal tissue was conducted following Kiernan's method. The tissue sections were stained using Hematoxylin and Eosin (HE) staining. Histological observation of *C. gariepinus* intestinal tissue was carried out using an Olympus 1 × 51 light microscope at 400x magnification, equipped with an OptiLab Advance Plus microscope camera. Goblet cell counts were performed by examining three sections of intestinal epithelium, each measuring 1000 µm (1 mm) in length. Cell counts were taken from four clockwise fields of view [44] using the OptiLab Viewer 4 application.

### 2.6. Fish Digestive Enzymes Analysis

Fish digestive enzymes measured in this study included amylase, protease, and lipase, using samples derived from intestinal organs. In the initial step, the intestinal samples were cut, weighed, and mixed with phosphate-buffered saline (PBS) at a 1:9 ratio. The intestines were then homogenized under cold conditions, followed by sonication and centrifugation to obtain the supernatant. Enzyme levels in the intestinal supernatant and serum were measured as follows: amylase levels using the Fish Amylase ELISA Kit (BT Lab, Zhejiang, China), lipase levels using the Fish Lipase ELISA Kit (BT Lab, Zhejiang, China), and protease levels using the Fish Protease ELISA Kit (BT Lab, Zhejiang, China).

### 2.7. Data Analysis

The effects of DSP treatments on the bacterial counts and digestive enzyme levels of catfish were statistically analyzed using SPSS version 26. Data were tested for normality and homogeneity using the Kolmogorov–Smirnov test and Levene's test, respectively. All data were analyzed using two-way analysis of variance (ANOVA) at a 5% significance level. When significant effects were detected, Duncan's multiple range test (DMRT) was applied for pairwise comparisons. Parameters related to DNA sequencing results and intestinal physiology were analyzed descriptively.

## 3. Results

### 3.1. Conventional Counting of Cultured Bacteria

The results demonstrated an increasing trend in the total number of microbes and lactic acid bacteria (LAB) in the catfish intestines, corresponding to the rising concentrations of DSP (Table 2). However, a decrease in total microbial count was observed in Treatment D (15% DSP in infected catfish) compared to Treatments A, B, and C. Among infected catfish, the LAB count was significantly higher in Treatment D than in Treatments A, B, and C. Meanwhile, there were no significant differences in the total microbial counts among the infected groups receiving 5%, 10%, and 15% DSP. In healthy catfish, supplementation with 15% DSP (Treatment H) resulted in significantly higher counts of both LAB and total microbes.

### 3.2. Gut Bacterial Profile Based on Metagenomic Analysis of Catfish Intestines

**Figure 1:**
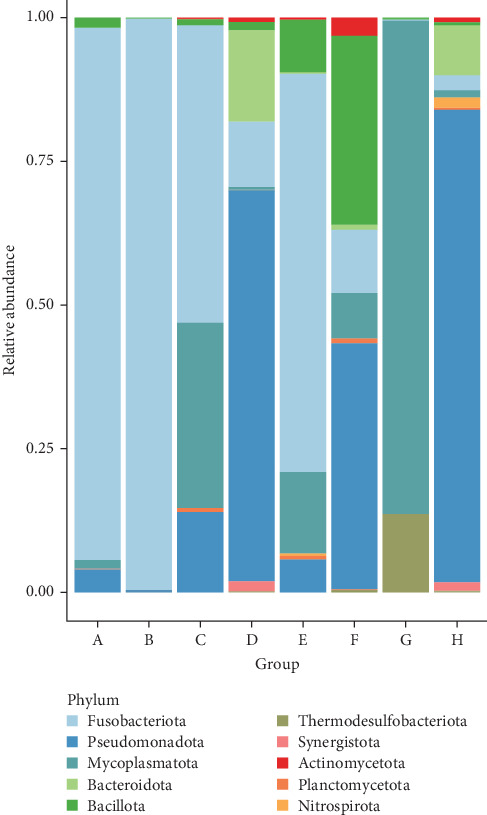
Top 10 phyla in each treatment and their relative abundance.

**Figure 2:**
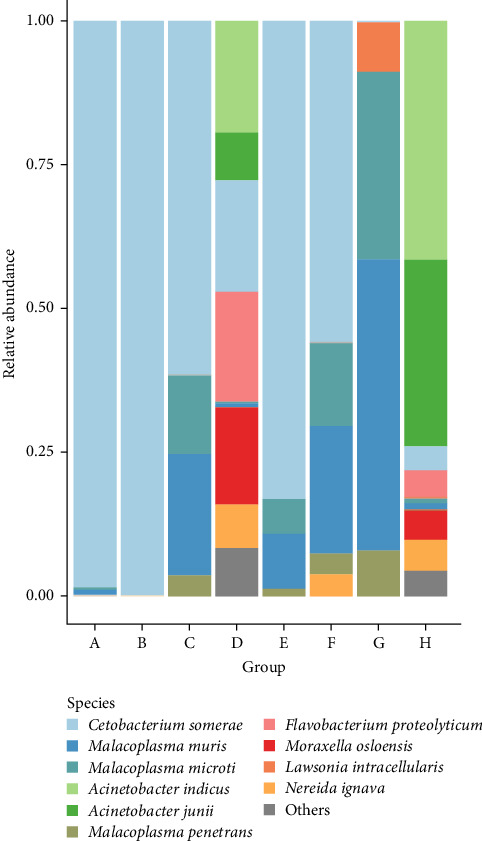
Top 10 species in each treatment and their relative abundance.

The screening of microbial components revealed the 10 most abundant phyla: Fusobacteria, Pseudomonadota, Mycoplasmatota, Bacteroidota, Bacillota, Thermodesulfobacteriota, Synergistota, Actinomycetota, Planctomycetota, and Nitrospirota (Figure 1). Catfish treated with 5% DSP (Treatment B) were predominantly colonized by the phylum Fusobacteriota, while Treatments G and H showed higher abundances of Mycoplasmatota and Pseudomonadota, respectively. Notably, Treatment F (10% DSP for healthy catfish) was characterized by a major presence of Bacillota and Pseudomonadota. Across all Treatments, *Cetobacterium somerae* was detected and was the dominant microbe in Treatments A, B, C, D, E, and F (Figure 2). In Treatment H, *Acinetobacter indicus* and *Acinetobacter junii* were highly abundant. Meanwhile, Treatment G exhibited a more balanced distribution among the top 10 microbial taxa.

**Figure 3:**
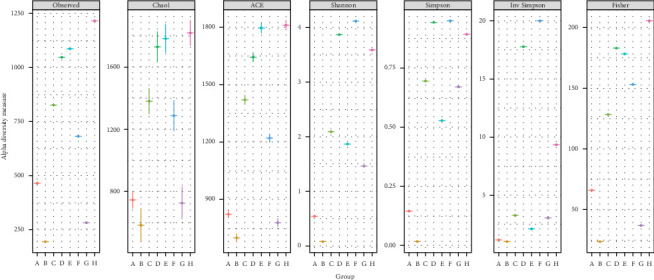
Comparison of alpha diversity across treatments. This analysis includes measures of species richness—represented by Chao1 and observed species—and species evenness—represented by the Shannon and Simpson indices. Alpha diversity reflects the mean species diversity within sites or habitats at a local scale.

**Figure 4:**
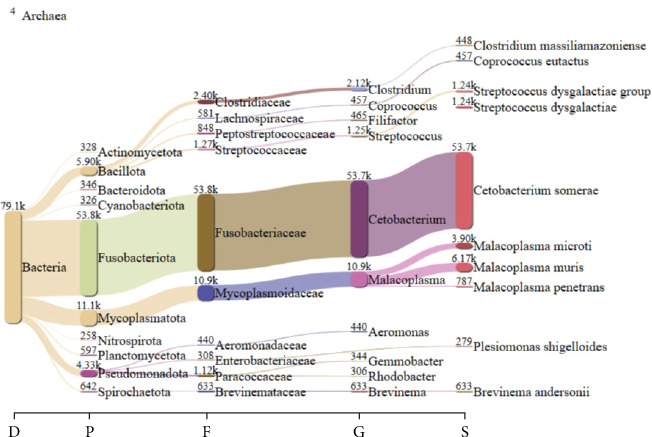
Sankey diagram of catfish treated without infection and 0% DSP supplementation (Treatment E). In this diagram, the width of each arrow is proportional to the quantity, illustrating hierarchical changes over time between nodes.

**Figure 5:**
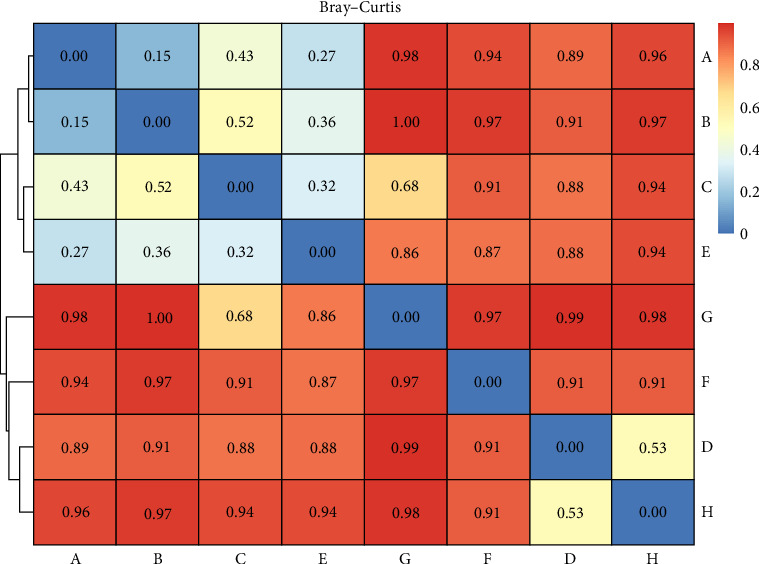
Microbial community in the catfish gastrointestinal tract based on the Bray–Curtis index.

**Figure 6:**
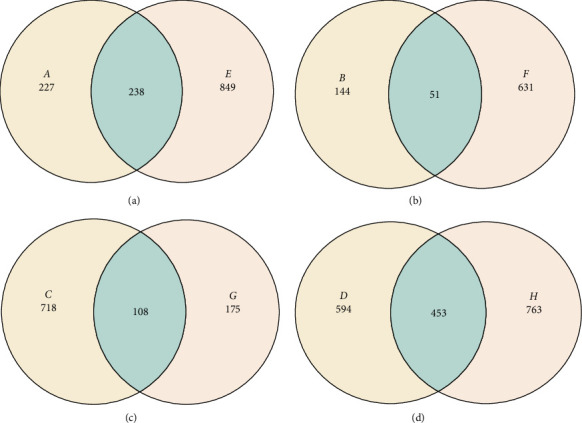
Venn diagram showing the number of unique and shared operational taxonomic units (OTUs) between treatment groups. The overlapping areas indicate the number of OTUs shared between groups, while the non-overlapping areas represent unique OTUs. (a) OTUs between A and E. (b) OTUs between B and F. (c) OTUs between C and G. (d) OTUs between D and H. Each circle represents the bacterial community composition of a specific group: A = infected fish without probiotic supplements, B = infected fish with 5% DSP, C = healthy fish with 5% DSP, D = infected fish with 15% DSP, E = healthy control, F = infected control, G = healthy fish with 10% DSP, H = healthy fish with 15% DSP.

Metagenomic analysis revealed that the highest microbial diversity was found in Treatments D and H, both of which involved catfish (infected and healthy, respectively) supplemented with 15% DSP (Figure 3). The alpha diversity of the intestinal microbiota was evaluated using Chao1, observed species, Shannon, and Simpson indices. Meanwhile, the Sankey diagram (Figure 4) illustrates the microbial composition dynamics in the control group (0% DSP, uninfected catfish). In contrast, the lowest diversity was observed in Treatments B and G, corresponding to DSP concentrations of 5% and 10%, respectively. Further analysis of the number of microbial genera indicated that treatments supplemented with 15% DSP had a higher genus count compared to the other groups (Figure 5). Conversely, Treatment B (5% DSP) exhibited the lowest genus count among all treatments. Although microbial compositions varied across treatments, some taxa were shared among them (Figure 6). The highest number of shared microbial taxa (453 records) was found in the 15% DSP groups, while only 51 shared microbes were identified in the 5% DSP groups.

According to Figure 5, in the infected fish groups receiving low DSP supplementation, the primary factor influencing microbial variability was the administration of *A. hydrophila*. However, at higher DSP concentrations, microbial profiles tended to converge, and the impact of pathogen infection became less pronounced. Therefore, the DSP dose appeared to be the more dominant factor in shaping microbial variability.

### 3.3. Gut Physiology

**Figure 7:**
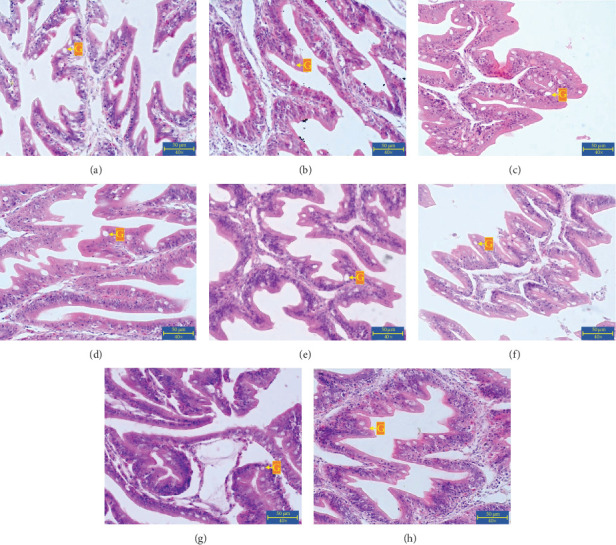
Microphotograph of the intestine of *C. gariepinus*. (a) (*A. hydrophila* infection + 0% DSP), (b) (*A. hydrophila* infection + 5% DSP), (c) (*A. hydrophila* infection + 10% DSP), (d) (*A. hydrophila* infection + 15% DSP), (e) (*A. hydrophila* infection + 0% DSP), (f) (*A. hydrophila* infection + 5% DSP), (g) (*A. hydrophila* infection + 10% DSP), and (h) (*A. hydrophila* infection + 15% DSP); G, Goblet cells.

According to Figure 7, the number of intestinal goblet cells in Group A (21.58 ± 1.63 cells/1000 µm) was higher compared to Group E (13.35 ± 1.03 cells/1000 µm). Further analysis of goblet cell counts across groups revealed varying results. Group F exhibited 11.34 ± 0.85 cells/1000 µm, while Group H and G had 10.03 ± 2.18 and 9.25 ± 0.94 cells/1000 µm, respectively. Groups B (9.97 ± 1.30) and C (9.27 ± 2.63) showed slightly higher goblet cell counts than group D (7.81 ± 1.51), but all were lower than Group A. However, statistical analysis indicated that the differences in goblet cell numbers among groups were not significant (*p*  > 0.05).

### 3.4. Digestive Enzyme Levels

The levels of digestive enzymes, including amylase, lipase, and protease, were measured after the 42-day catfish rearing period. A significant difference in protease levels was observed between the 5% probiotic treatment Group (B) and the 10% and 15% treatment groups, namely C (10% DSP, infected), D (15% DSP, infected), G (10% DSP, noninfected), and H (15% DSP, noninfected). Overall, amylase levels were higher in the healthy fish group compared to the pathogen-infected group. A similar trend was observed for lipase levels. However, among the infected fish, those treated with 15% DSP exhibited higher levels of both lipase and protease. Digestive enzyme levels measured in fish intestinal organs are in Table 3.

## 4. Discussion

Catfish possess a natural microflora that resides on their skin, gills, and in their intestines [45]. However, the composition of this microflora frequently changes in response to environmental fluctuations and variations in water quality [46]. To support the growth and development of catfish, various probiotics are commonly added as feed supplements [47]. Probiotics are live microorganisms that are beneficial and capable of colonizing the host's gastrointestinal tract [48, 49]. They help maintain microbial balance and inhibit the proliferation of pathogens in the gastrointestinal system of fish [14]. The use of probiotics in aquaculture has also been shown to improve digestion and enhance nutrient absorption by modulating the gut microbiota [17, 48]. Among the most widely used probiotic bacteria are *B. subtilis* and *L. casei* [28, 50, 51].

Research on DSP, consisting of *L. casei* and *B. subtilis*, has been conducted in catfish, and the results demonstrated beneficial effects on growth performance and the immune system of catfish infected with *A. hydrophila* [27]. Similarly, a study by Luo et al. [34] also demonstrated that the addition of a dual probiotic strain combination—*B. subtilis* and *L. plantarum*—improved growth, enhanced digestive enzyme activity (including total protease, amylase, lipase, and trypsin), and increased bacterial diversity in the gastrointestinal tract of grass carp (*Ctenopharyngodon idella*).

Even within the same fish species, differences in lifestyle can lead to the presence of distinct types and quantities of bacteria [52]. These variations are influenced by the specific probiotic treatments applied. The number of gastrointestinal bacteria tends to increase with higher probiotic dosages. However, traditional methods for estimating bacterial quantity and diversity—such as culturing on growth media—are considered less accurate, as some bacteria cannot be recultured. Consequently, these methods may not accurately reflect the true bacterial abundance and diversity. Therefore, in this study, bacterial profiling of the gastrointestinal tract was not only supported by conventional culturing methods but also performed using NGS to obtain a more comprehensive overview.

In the current study, the total bacterial counts isolated from the intestines of catfish treated with probiotics ranged from 10^3^–10^5^ CFU/g. This count was higher than that observed in the control group. As the concentration of probiotics increased, the total bacterial load in the gut also increased. This is likely due to a greater number of probiotic bacteria surviving and colonizing the host's digestive tract. However, these values are still relatively low compared to those reported in previous studies. For instance, Wu et al. [52] reported that the microbial population in the gastrointestinal tract of fish ranges from 10^7^–10^8^ CFU/g.

Aini et al. [50] conducted a study to determine the number of bacteria in the gastrointestinal tract of catfish randomly sampled from local fish farmers in Surabaya, Indonesia. They reported bacterial counts of 8.7 × 10^4^ CFU/g for total bacteria and 1.2 × 10^5^ CFU/g for LAB [50]. Similarly, Jimoh et al. [53] isolated microbes from the gastrointestinal tract of catfish collected from rivers in Nigeria, with bacterial counts reaching 6.5 × 10^5^ CFU/g [43]. Variations in bacterial counts within the same species can be attributed to several factors, including differences in habitat, environmental conditions, genetic background, and diet [53].

With the addition of DSP, both the total bacterial count and the number of LAB increased compared to the control group. This is likely due to the ingestion of feed supplemented with DSP, which allows the probiotics to enter the catfish's digestive tract. DSP is expected to proliferate and colonize the intestinal mucosa of catfish, thereby enhancing its probiotic effects [34]. The presence of DSP in the feed, particularly at the highest concentration, may account for the increased number of LAB. In fish infected with pathogens, bacterial counts were lower compared to those in uninfected groups. This may be due to competition among bacteria for nutrients and colonization sites within the gut [54].

In recent years, numerous studies have been conducted to investigate the composition of normal microflora in catfish. These findings can be further utilized to identify the probiotic potential of specific bacterial strains. Understanding the microbial profile of the fish gastrointestinal tract also reveals that some opportunistic pathogens may be part of the normal gut flora [55]. A stable gut microflora plays a crucial role in enhancing the fish's natural resistance to infections caused by pathogenic bacteria in the digestive system [32].

Interactions between microbes and fish gut tissues are dynamic. Accordingly, microbes can be categorized based on the duration of their residence in the gastrointestinal tract, as either transient or persistent bacteria [56]. Transient bacteria typically enter the fish gut through food intake. However, they do not persist for long, as the intestinal wall is already colonized by persistent bacteria. These resident bacteria have long been established in the fish gastrointestinal tract and maintain a close symbiotic relationship with the host [57].

NGS analysis revealed that *B. subtilis* and *L. casei* were not detected in the gastrointestinal tract of catfish subjected to any of the DSP treatments. This indicates that DSP bacteria are likely do not permanently colonize in the gut of catfish. We acknowledge this as a limitation and interpret it to indicate transient passage rather than persistent colonization. Many probiotic strains are generally considered transient in the gastrointestinal tract, with their persistence depending on continuous supplementation, host–microbe interactions, and environmental conditions. The absence of *L. casei* and *B. subtilis* in NGS profiles suggests that these strains may act as transient members of the gut community rather than establishing permanent colonization. Nonetheless, transient probiotics are still able to exert significant effects on gut health through various mechanisms, including competitive exclusion of pathogens, secretion of extracellular enzymes and metabolites that improve feed digestibility, modulation of resident microbiota, and stimulation of host immune responses. Several studies in aquaculture and terrestrial animals have also reported that probiotic strains can provide measurable benefits without long-term colonization [58, 59]. Nevertheless, even if they do not form colonies, the presence of DSP still contributes to feed fermentation and enhances feed digestibility. Moreover, DSP appears to stimulate the secretion of digestive enzymes, improving digestive efficiency.

Thus, lack of detection does not necessarily negate functional effects. This view is consistent with broader probiotic literature and aquaculture-focused syntheses, which report health, growth, and resilience benefits even when administered strains do not persist long-term. Nevertheless, colonization potential is strain- and host-dependent; autochthonous fish isolates may display greater persistence than non-host strains, and clear engraftment has been observed in some fish models for selected Bacillus lineages. Future work should therefore combine strain-specific tracking (e.g., qPCR/shotgun metagenomics with unique markers) with functional readouts to disentangle transient versus persistent mechanisms [59, 60].

The microbial composition of the fish gut is strongly influenced by the diet [61, 62]. Fish feed supplemented with DSP results in a different microbial diversity compared to feed without DSP. In both infected and uninfected fish groups that received feed without DSP, the dominant phyla were Fusobacteria and Pseudomonadota. In contrast, the groups treated with varying concentrations of DSP showed noticeable shifts in microbial composition. The predominant phyla found in the intestines of these fish included Pseudomonadota, Bacillota, and Bacteroidota. At a DSP concentration of 10%, Mycoplasmatota was the dominant phylum.

Previous research on channel catfish reported that the major bacterial phyla present in the gut were Firmicutes, Proteobacteria, and Bacteroidetes [63]. Additionally, the most abundant species observed in Treatments A and B was *Cetobacterium somerae*, which belongs to the phylum Fusobacteria. This bacterium is known for its ability to produce vitamin B_12_ and is commonly found in the gastrointestinal tract of freshwater fish [61].

The observed stability in microbial variability among fish infected with *A. hydrophila* may be attributed to the pathogen's role as a vaccine-like agent in catfish that received probiotic supplementation. The administration of probiotics appears to have enhanced the immune system, thereby preventing adverse health effects despite the presence of pathogens. A study conducted by Mulia et al. [64] demonstrated that administering an *A. hydrophila* GPl-04 vaccine through feed increased disease resistance and improved growth performance in catfish.

The use of NGS to characterize the microbial profile of the fish gut is considered highly advantageous, given that many bacterial species cannot be cultured or visualized under a microscope. It is estimated that only 0.01%–1% of the total bacterial population can be cultured on growth media [65]. Variations in the microbial profiles of catfish intestines were observed across treatments with different concentrations of DSP. The composition of gut microbiota in fish is influenced by both biotic and abiotic factors. Biotic factors include genotype, physiological status, disease pathology, and lifestyle, while abiotic factors encompass environmental conditions [46]. Some studies have even reported that microbes present in the surrounding water are also commonly found in the gastrointestinal tracts of fish [66].

Another limitation of the present study is that the administered probiotics were not detected in the gut microbiota profiles, suggesting transient rather than permanent colonization. In addition, a limitation of the present metagenomic analysis is that it was restricted mainly to phylum-level changes, whereas genus-level dynamics and functional profiles could provide more detailed insights into the specific taxa and pathways influenced by dietary probiotics. Future studies employing more comprehensive taxonomic and functional analyses are warranted to further elucidate the mechanisms underlying microbiota modulation in catfish.

The fish gut is a component of the digestive tract that functions in nutrient absorption and the production of various digestive enzymes. These enzymes work synergistically with microorganisms such as bacteria, fungi, and yeasts that colonize the digestive system to form the gut microbiota. The microbiota lives in symbiosis with the gut, providing metabolic benefits and protecting the host from pathogenic infections [67]. Additionally, the gut microbiota can be externally supplemented through the administration of beneficial microorganisms, commonly known as probiotics.

Histologically, the fish intestine is composed of four layers: the mucosa, submucosa, muscle layer, and serosa [68]. Among these, the mucosal layer is the only one directly exposed to food and the external environment, making it particularly vulnerable to pathogen invasion. As a result, the mucosal layer is specialized and functionally distinct from other layers of the gastrointestinal tract [69]. Within this layer, specialized secretory cells known as goblet cells produce mucin, a gel-like substance that protects the intestinal lumen [70]. According to Sveen et al. [71], mucin secretion responds to dietary changes, pathogen exposure, and handling stress, serving as an indicator of fish health. Histologically, goblet cells appear as transparent, rounded cells located within the epithelial layer of the villous columnar structure of the intestinal mucosa [72], as illustrated in Figures 7.

This finding aligns with the study by Purba et al. [73], which reported a substantial number of goblet cells in the intestines of fish following *A. hydrophila* infection. Infection by this pathogenic bacterium stimulates the secretion of excess interleukin-9 (IL-9), leading to increased goblet cell proliferation [74]. However, statistical analysis indicated that these variations were not significant (*p* > 0.05). This suggests that, although numerical differences were detected, the probiotic supplementation and pathogen challenge did not result in measurable changes in goblet cell counts under the conditions of this study. This may be due to the *A. hydrophila* infection being administered intraperitoneally, which may not have had a direct or maximal impact on the number of goblet cells in the intestines of *C. gariepinus*. The lack of significant differences may be related to the infection route, as the intraperitoneal administration of *A. hydrophila* may have had limited direct effects on the intestinal mucosa. Furthermore, goblet cell numbers alone may not fully capture the complexity of intestinal responses to probiotics and pathogens. Additional histological parameters, such as villus morphology, mucosal integrity, and inflammatory cell infiltration, would provide a more comprehensive assessment of intestinal health.

Oral administration of probiotics enables interaction with intestinal epithelial cells (IECs) in the lamina propria via Toll-like receptors, which mediate immune system activation. Probiotics also enhance the intestinal barrier by increasing the number of goblet cells, which secrete large amounts of mucin, thereby reinforcing the mucus layer in the lamina propria [75, 76]. The addition of DSP, as an external bacterial stimulus recognized by the gut as an antigen, promotes a defensive mucosal response by increasing goblet cell numbers and mucin secretion [77].

Treatments A, B, and C showed no significant differences in goblet cell counts when compared across groups. The lower number of goblet cells in Group D may be attributed to infection by pathogenic bacteria, which can damage the mucosal layer in the intestinal lamina propria, leading to structural changes in goblet cells and a reduction in mucin secretion [78]. Conversely, the higher number of goblet cells in Groups B and C compared to the normal group A is consistent with the findings of Gāliņa et al. [79], who reported that probiotic supplementation can stimulate goblet cell proliferation in the intestinal lamina propria. An increase in goblet cell proliferation leads to greater mucin secretion, thereby thickening the mucus layer and enhancing protection of the intestinal epithelium against harmful agents [80]. The lack of significant difference overall suggests that *A. hydrophila* infection did not have a measurable impact on goblet cell numbers in the intestines of *C. gariepinus*. This may be due to differences in the infection route, as the intraperitoneal administration used in this study may have limited direct effects on the intestinal mucosa. It should be noted that the histological assessment in this study was limited to goblet cell counts as an indicator of mucin secretion and gut protection. While useful, this single parameter does not fully capture the complexity of intestinal health. Additional histological features such as villus architecture, mucosal integrity, and inflammatory cell infiltration should be examined in future research to provide a more comprehensive evaluation of the effects of dietary probiotics on the catfish intestine.

Administering probiotics to fish through feed has been shown to increase the production of digestive enzymes such as amylase, lipase, chitinase, and protease [81, 82]. The concentration of these enzymes reflects the physiological effectiveness of nutrient metabolism and influences the efficiency of feed utilization to support fish growth and development [83, 84]. Jang et al. [81] demonstrated that probiotics improve digestion by enhancing enzyme production, increasing both the abundance and diversity of beneficial microbial populations in the gastrointestinal tract, boosting microbial enzymatic activity, and promoting microbial balance, all of which contribute to greater nutrient absorption and optimal growth rates. However, it remains unclear whether these enzymes are produced directly by probiotics, stimulated via modulation of the fish's own enzyme synthesis and secretion mechanisms, or through a combination of both processes [30]. The provision of DSP in fish feed has been found to enhance enzyme production in the gastrointestinal tract of catfish. According to Zhang et al. [57] probiotics can induce the secretion of exogenous enzymes, though these contribute only a small fraction of the total digestive enzyme content. Nonetheless, probiotics can significantly stimulate the production of endogenous enzymes synthesized by the fish itself [57].

Afrilasari et al. [85] reported that adding *B. megaterium* PTB 16 to catfish infected with *A. hydrophila* improved digestive enzyme production compared to the control group. Similarly, Lawal et al. [55] found that supplementation with *B. subtilis* in catfish fry increased protease production after 8 weeks of feeding. *B. subtilis* has been widely recognized for its ability to produce extracellular enzymes and its resilience under extreme temperatures and limited water conditions [31, 51]. In addition, Wang et al. [28] showed that the inclusion of *L. casei* K17 in the diet of largemouth bass (*Micropterus salmoides*) significantly enhanced digestive enzyme activity.

The combination of *B. subtilis* and *L. casei* has also been shown to increase digestive enzyme production. In this study, administration of DSP improved digestive enzyme synthesis in catfish compared to the control group. Among fish infected with *A. hydrophila*, the overall digestive enzyme levels were lower than those in uninfected fish. Digestive enzymes play a critical role in breaking down and assimilating feed nutrients, thereby improving feed efficiency. Research by Aini et al. [27] confirmed that DSP supplementation led to a lower feed conversion ratio (FCR) and better growth performance in catfish compared to fish that did not receive DSP.

## 5. Conclusions

The addition of DSP (containing *L. casei* and *B. subtilis* in a 1:1 ratio) at various concentrations in the feed may play an important role in increasing the number and diversity of gastrointestinal bacteria, improving intestinal physiology, and enhancing the levels of digestive enzymes (i.e., amylase, lipase, and protease) in catfish infected with *A. hydrophila*. The optimal DSP concentrations for improving all parameters were 10% and 15%.

## Figures and Tables

**Table 1:** Description of treatment groups and experimental replicates.

*Aeromonas hydrophila*	DSP concentration (%)	Treatment group and repetition
Infected	0	A (A1, A2, A3)
	5	B (B1, B2, B3)
	10	C (C1, C2, C3)
	15	D (D1, D2, D3)

Not Infected	0	E (E1, E2, E3)
	5	F (F1, F2, F3)
	10	G (G1, G2, G3)
	15	H (H1, H2, H3)

**Table 2:** Total microbial and lactic acid bacteria (LAB) counts in the intestines across eight catfish treatments.

Treatments	Lactic acid bacteria (LAB) count (CFU/mL)	Total intestinal bacterial count (CFU/mL)
A	3.54 ± 0.34^a^	4.96 ± 0.61^ab^
B	4.39 ± 0.24^b^	5.47 ± 0.30^bc^
C	4.08 ± 0.15^ab^	6.02 ± 0.41^c^
D	6.20 ± 0.38^d^	5.65 ± 0.25^bc^
E	3.94 ± 0.59^ab^	4.45 ± 0.27^a^
F	4.93 ± 0.19^c^	4.66 ± 0.32^a^
G	5.16 ± 0.23^c^	6.04 ± 0.44^c^
H	6.22 ± 0.05^d^	6.79 ± 0.47^d^

*Note:* The values with different superscript letters in a column are significantly different (*p* < 0.05).

**Table 3:** Digestive enzyme levels measured in fish intestinal organs.

Treatments	Amylase (ng/mL)	Lipase (ng/mL)	Protease (ng/mL)
A	92.12 ± 3.41^ab^	104.40 ± 12.86^a^	105.30 ± 12.48^d^
B	84.37 ± 10.55^a^	91.19 ± 14.69^a^	114.34 ± 4.02^c^
C	97.42 ± 2.52^b^	113.03 ± 3.00^a^	84.46 ± 0.95^b^
D	100.91 ± 2.16^b^	138.02 ± 8.99^b^	86.53 ± 8.27^b^
E	92.66 ± 6.25^ab^	109.57 ± 21.19^a^	60.75 ± 9.42^a^
F	148.08 ± 3.75^d^	173.79 ± 12.84^c^	87.24 ± 6.04^b^
G	121.09 ± 5.58^c^	170.21 ± 10.01^c^	92.66 ± 5.71^b^
H	98.88 ± 10.79^b^	104.4015 ± 12.86^a^	70.84 ± 4.05^a^

*Note:* The values with different superscript letters in a column are significantly different (*p*  < 0.05).

## Data Availability

The data that support the findings of this study are available from the corresponding author upon reasonable request.
